# Residual ground-water levels of the neonicotinoid thiacloprid perturb chemosensing of *Caenorhabditis elegans*

**DOI:** 10.1007/s10646-017-1826-z

**Published:** 2017-06-22

**Authors:** Hannah Hopewell, Kieran G. Floyd, Daniel Burnell, John T. Hancock, Joel Allainguillaume, Michael R. Ladomery, Ian D. Wilson

**Affiliations:** Department of Applied Sciences, Faculty of Health and Applied Sciences, University of the West of England, Bristol, Frenchay Campus, Coldharbour Lane, Bristol, BS16 1QY UK

**Keywords:** Neonicotinoid, Thiacloprid, Chemosensing, Nematodes, *Caenorhabditis elegans*

## Abstract

This study investigated the neurological effects of residual ground-water levels of thiacloprid on the non-target organism *Caenorhabditis elegans*. Nematodes treated with thiacloprid showed a dose-dependent and significantly increased twitch response at concentrations above 50 ng mL^−1^ that disabled their forward locomotion in liquid culture. In comparison with untreated controls, 10 ng mL^−1^ thiacloprid perturbed the chemosensory ability of *C. elegans* such that the nematodes no longer demonstrated positive chemotaxis towards a NaCl chemo-attractant, reducing their chemotaxis index from +0.48 to near to zero. Nematodes also exhibited a locomotion characteristic of those devoid of chemo-attraction, making significantly more pirouetting turns of ≥90° than the untreated controls. Compared to the untreated controls, expression of the endocytosis-associated gene, *Rab-10*, was also increased in *C. elegans* that had developed to adulthood in the presence of 10 ng mL^−1^ thiacloprid, suggesting their active engagement in increased recycling of affected cellular components, such as their nAChRs. Thus, even residual, low levels of this less potent neonicotinoid that may be found in field ground-water had measurable effects on a beneficial soil organism which may have environmental and ecological implications that are currently poorly understood.

## Introduction

Thiacloprid is a member of the neonicotinoid family of systemic insecticides which act as agonists of the post-synaptic ligand operated ion channels of the nervous system, the nicotinic acetylcholine receptors (nAChRs) (Matsuda et al. 2005, 2009). Irreversible binding of neonicotinoids to nAChRs induces muscular over-stimulation, paralysis and death (Yamamoto 1999; Vo et al. 2010; Easton and Goulson 2013). Unlike nicotine, the neonicotinoids are characterised by a common pharmacophore containing either a nitroimine, nitromethylene or cyanoimine group which confers, supposedly, selective toxicity because the specific amino acids of the loop structures of the ligand binding domains that enable interaction with these compounds are thought to be exclusively present in insect nAChRs (Tomizawa and Casida 2005). Agronomically, as systemic insecticides, neonicotinoids are usually applied as a seed dressing and are taken up during subsequent germination and growth, rendering the plant persistently toxic to any chewing or sucking insect.

In 2013 the European Food Safety Authority (EFSA) enforced a 2 year Europe wide ban on the use of the three neonicotinoids, imidacloprid, thiamethoxam and clothianidin because of their suspected involvement in bee colony collapse disorder. Several studies outlined the possible effects of these pesticides on bee populations as a result of exposure to contaminated pollen, nectar and dust from treated crops (Krupke et al. 2012; Blacquière et al. 2012; Sanchez-Bayo and Goka 2014). Exposure to neonicotinoids was shown to reduce queen survival rate and colony size and to disrupt the highly evolved navigational mechanisms employed by bees during their foraging and return to the hive (Fischer et al. 2014; Williams et al. 2015). Additionally, more recent concerns have been highlighted surrounding the effects of neonicotinoids on non-target, invertebrate organisms (Pisa et al. 2015).

Despite these concerns, thiacloprid escaped the ban due to its reported lower insect toxicity (Iwasa et al. 2004). However, other studies have suggested it may be more toxic to fish than many of the other neonicotinoids (Schmuck 2001). Thiacloprid is also considered one of the more persistent neonicotinoids in the environment. Since it does not absorb light beyond 290 nm (Peña et al. 2011), it is not readily subjected to sunlight-induced photolysis (Gupta et al. 2008) and with its chemical stability, may take considerable time to degrade in the environment (Černigoj et al. 2007). The half-life of thiacloprid is stated as being 19.1 days in soil, but may be considerably longer in anaerobic soils and it has been suggested that, with repeated use on water retentive soils, the groundwater residue concentrations of this neonicotinoid, as for imidacloprid, may similarly reach between 18 to 60 ng mL^−1^ depending on the application rate (Goulson 2013), which is within the range at which it could be toxic to susceptible organisms (Langer-Jaesrich et al. 2010). Consequently, this study investigated the effects of environmentally realistic levels of thiacloprid on the non-target, model organism *Caenorhabditis elegans*, a soil-borne nematode.

## Materials and methods

### Growth, culturing and treatment of *C. elegans* with thiacloprid

The Bristol N2 strain of *C. elegans* was gratefully received from Prof. Patricia Kuwabara at Bristol University, Bristol, UK and aseptically maintained at 21 °C in the dark either on nematode growth media (NGM) agar plates spread with a lawn of the slow-growing, uracil auxotrophic *Escherichia coli* strain, OP50, as a food source or in shaking (20 rpm), liquid, M9 buffer culture, again using the same strain of *E. coli* as food (Stiernagle 2005). Thiacloprid (C_10_H_9_ClN_4_S) was obtained from Sigma-Aldrich (PESTANAL analytical standard grade, suitable for HPLC and >99.9% pure). In order to determine the immediate and short term physical effects of thiacloprid on *C. elegans*, nematodes were exposed for 1 h in liquid culture to a range of concentrations (0.1 ng mL^−1^ to 10 µg mL^−1^) of the pesticide. To assess the effect of the pesticide on mortality, after the incubation period, individual treatments were scored for their kill percentage by counting the proportion of the nematodes that had attained the motionless, straightened, rod shape appearance which is characteristic of dead *C. elegans* in liquid media.

### Thiacloprid-induced twitch assays

Mixed developmental stage populations of *C. elegans* were gently washed from NGM plates using S basal media (Stiernagle 2005) and using the same buffer throughout were washed three times by low speed centrifugation (100 x g, 30 s, 21 °C) to remove contaminating *E. coli* and were finally resuspended at 1000 nematodes mL^−1^. Replicate aliquots containing 50 *C. elegans* were then mixed in 96 well plates with buffer containing thiacloprid to give final volumes of 100 µL with a range of thiacloprid concentrations between zero and 10 µg mL^−1^. Nematodes were incubated with the pesticide for 30 min at 21 °C and their mortality and twitch movements were then observed under a binocular microscope and recorded using video capture at 20 frames s^−1^.

### Chemotaxis assays

**Fig. 1:**
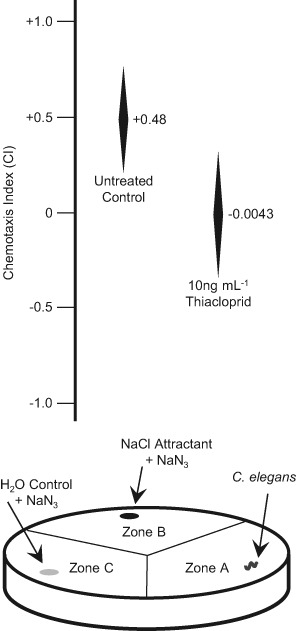
Thiacloprid altered the chemosensing of NaCl by *C. elegans*. Six replicate samples of mixed growth stage populations of ≈150 *C. elegans* (N_total_) were treated +/−10 ng mL^−1^ thiacloprid for 1 h at 21 °C in S basal buffer before being applied to zone A of individual chemotaxis plates (represented above). The nematodes were allowed to migrate on the plates for a further 1 h before the numbers present in each zone were counted under a microscope. The mean numbers of the nematodes present in zones B and C (N_B_ and N_C_) along with the corresponding 95% confidence intervals (95% Conf. Int.) around the means were calculated in each case. Significant differences (*P* < 0.05), or not (*P* > 0.05), in the numbers of nematodes migrating to each zone were determined by one way ANOVA and the chemotaxis index (CI) ranges shown were then calculated at the 95% confidence level using the formula CI = (N_B_ − N_C_)/N_total_. Thus, the upper CI value of each range arose from [(N_B_ + 95% Conf. Int.) − (N_C_ − 95% Conf. Int.)]/N_total_ and the lower CI value from [(N_B_ − 95% Conf. Int.) − (N_C_ + 95% Conf. Int.)]/N_total_. The mid-point CI values are indicated in each case

Replicate chemotaxis assays were performed according to Saeki et al. 2001 using NaCl as the chemo-attractant. Mixed developmental stage populations of *C. elegans* were collected from NGM plates as before and were treated in liquid culture +/−10 ng mL^−1^ thiacloprid for 1 h at 21 °C before their application to chemotaxis plates (Fig. 1). Replicate 8 cm diameter NGM agar plates were divided into three equal, wedge shaped zones and ≈150 nematodes, both treated and untreated control, were placed in zone A and given the choice of migrating towards either a NaCl attractant spot in zone B or a control spot in zone C. Both the control and attractant spots also contained a small amount of NaN_3_, applied as 1 µL of a 0.5 M solution just before plates were used, which effectively immobilised the nematodes that reached either position.

Nematodes were allowed to migrate for 1 h at 21 °C on the plates before their positions were recorded using a binocular microscope. Chemotaxis indices were then calculated as described (Saeki et al. 2001). Chemotaxis plates were also photographed after the 1 h migration in order to visualise the tracks left by individual *C. elegans* and the number of pirouetting turns of ≥ 90° that had been made by the nematodes was recorded.

### Developmental synchronisation of *C. elegans*

Eggs were collected from adult hermaphrodite *C. elegans* according to Stiernagle (2005) and were hatched on NGM agar plates without a food source. Hatched larvae were thus, maintained at 21 °C for 24 h to allow their synchronous development to the L1 stage before their transfer to *E. coli* OP50 NGM agar plates +/−10 ng mL^−1^ thiacloprid. Larvae were then allowed to develop for a further 48 h to young adult stage, were washed from plates using S-basal buffer as before and subsequently purified away from contaminating *E. Coli* by centrifugal flotation on a 30% (w/v) sucrose density cushion (Stiernagle 2005). The nematodes were aspirated from the top of the sucrose cushion and were washed with an excess of buffer, pelleted by centrifugation and the pellets were immediately frozen in liquid nitrogen.

### RT-PCR analysis

According to the manufacturer’s instructions, total RNA was extracted from individual frozen pellets of *C. elegans* using TRI Reagent® (Sigma-Aldrich, UK) and was subsequently treated with DNaseI (Ambion®, Thermo Fisher Scientific, UK) to remove contaminating gDNA.

In final vols. of 20 µL and using the buffer supplied, individual 8 μg aliquots of the DNase-treated total RNA samples were subjected to anchored oligo-(dT)_18_ (0.5 μg)-primed reverse transcription for 90 min at 42 °C using 400 units of SuperScript® II reverse transcriptase (Thermo Fisher Scientific, UK) in the presence of 500 μM dNTPs, 5 mM DTT and 20 units of SUPERase • In™ RNase inhibitor (Thermo Fisher Scientific, UK). The reactions were subsequently heat inactivated at 65 °C for 15 min and the resulting cDNA solutions diluted 1 in 100 with SDW before their use in PCR analysis.

Using the buffer supplied, PCR reactions (10 min at 95 °C, then 35 cycles of 1 min at 95 °C, 1 min at 55 °C, 1 min at 72 °C and finally 15 min at 72 °C) were performed in a final vol. of 25 µL containing 10 µL of diluted cDNA template and 2.5 units of MyTaq™ HS DNA Polymerase (Bioline, UK) in the presence of 2.5 µM forward and reverse DNA oligonucleotide primers and 500 µM dNTPs. Gene specific DNA oligonucleotide primer sets used for PCR amplification were: *Ama-1* (Forward 5′-CAGTGGCTCATGTCGAGT-3′, Reverse 5′-CGACCTTCTTTCCATCAT-3′); *Ced-4* (Forward 5′-TCGACGAGATGTGTGATTTAG-3′, Reverse 5′-GTTTTCGGTTCACAAGACTTG-3′) (Shaham and Horvitz 1996); *Rab-10* (Forward 5′-CGAGTTGTGAGCAGAGAACG-3′, Reverse 5′-CCTCTGTGGTTGCACTGGATTCACC-3′). Following amplification, PCR products were separated by electrophoresis alongside DNA size markers (Bioline, UK) in 1 × TAE 1.5% (w/v) agarose gels containing 1 µg mL^−1^ EtBr and were visualised under UV illumination.

### Data analysis and bioinformatics

Where appropriate, data were assessed for normality and between data group heteroscedasticity by Shapiro-Wilk and Bartlett’s tests. Data were then analysed for overall treatment effect by using either one way ANOVA or Kruskal-Wallis tests depending on their parametric adherence or not. Post hoc significant differences between the means of groups were then determined by either 2-sample t tests, Tukey-Kramer tests or by Dunn’s test. In all cases statistically significant differences were determined at the 95% confidence level (*P* < 0.05 that the null-hypothesis was acceptable).

The blastp alogorithm (Altschul et al. 1990) was used to search the non-redundant protein sequences database at the National Centre for Biotechnology Information (NCBI) to identify sequences from other neonicotinoid-susceptible species that were highly similar to that of the neurotransmitter-gated ion-channel ligand binding domain found between residues 23 to 229 of the full length amino acid sequence of the *C. elegans* nAChR-α16A subunit (NP_505207). Multiple sequence alignments were performed using Clustal Omega (Sievers et al. 2011) at the European Molecular Biology Laboratory, European Bioinformatics Institute (EMBL-EBI) (Goujon et al. 2010) and neighbour joining phylogenetic tree analysis of aligned sequences was performed using Paup 4.0b10 (Swofford 2002).

## Results

### Thiacloprid altered the motility of *C. elegans*

**Fig. 2:**
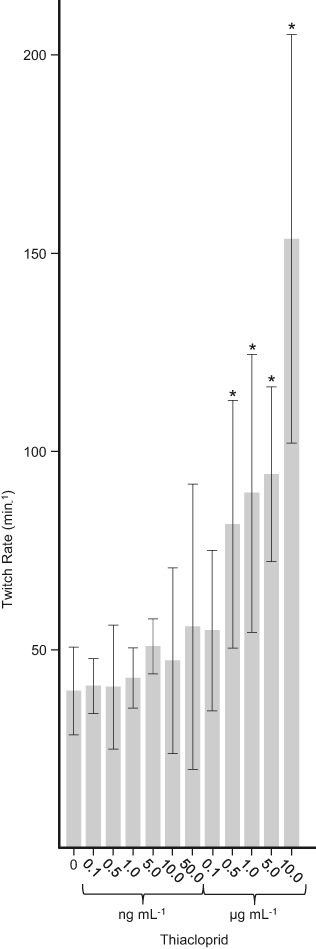
Elevated levels of thiacloprid induced an increased twitch response in *C. elegans*. Replicate (*n* = 3) experimental samples (50 nematodes per sample) of mixed growth stage populations of *C. elegans* suspended in S basal buffer were exposed to a range of concentrations of thiacloprid and in each case, the mean rates of twitching of the 150 individual nematodes were monitored under a microscope using video capture at 20 frames s^−1^. Results are presented as the mean twitch rate with errors shown as the 95% confidence intervals around the means for each concentration of thiacloprid used. *Indicates a twitch rate significantly increased above that of the control (*P* < 0.05 by Dunn’s test)

Based on the experience with the neonicotinoid imidacloprid, in which repeated applications of the pesticide have been shown to result in ground-water levels of 18–60 ng mL^−1^ (Goulson 2013), we decided to test a wide range of concentrations of thiacloprid upwards from 0.1 ng mL^−1^. Over the range of concentrations used there was no significant difference in the apparent mortality of the nematodes during the incubation period (data not shown). However, while the lower concentrations of thiacloprid appeared to have no immediate effect on the movement of the nematodes, those above 100 ng mL^−1^ caused a noticeable and significant increase in the rate of twitching of the nematodes compared to that of those in the untreated controls (Fig. 2) and also exaggerated the extent of the twitch movement itself. In the control S basal liquid cultures used here, individual *C. elegans* achieved forward locomotion by oscillating the full length of their bodies from side to side in apparent twitching movements at the rate of approximately 40 min^−1^. Here, a single twitch involved the nematodes progressing from a relatively straight body position through a curled position with head and tail in lateral proximity, followed by a subsequent return to a straight body appearance. Successive twitches involved the nematodes curling in the opposite direction to that of the immediately preceding twitch. At relatively high concentrations, between 0.5 to 10 µg mL^−1^, thiacloprid increased the rate of twitching of the nematodes to between approximately 75 to 155 min^−1^ respectively and also exaggerated the severity of the actual twitch movement so that the heads and tails of the nematodes became crossed at the height of the movement. At thiacloprid concentrations of ≥5 µg mL^−1^ forward movement of the nematodes also effectively ceased as they rapidly curled and uncurled in the same relative position.

### Thiacloprid altered NaCl chemo-sensing by *C. elegans*

Although concentrations of thiacloprid below 100 ng mL^−1^ did not appear to significantly alter the movement behaviour of *C. elegans*, its neurotoxic mode of action necessitated a more sensitive appraisal of its effects on the nematodes. One way to determine the extent of neurological perturbations in *C. elegans* is to monitor their ability to undertake chemotaxis towards a chemo-attractant, such as NaCl. Typically, such assays are routinely performed on agar plates divided into three or more zones. The nematodes, treated or otherwise, are applied to one zone and given free range to migrate towards other zones on the chemotaxis plate which may contain either different or competing chemo-attractants or which may operate as control zones.

Before their application to separate plates, replicate aliquots of *C. elegans* were treated +/−10 ng mL^−1^ thiacloprid for 1 h in liquid culture. This level of thiacloprid was chosen because it did not induce a significant increase in nematode twitching (Fig. 2) that may have potentially hampered their movement on the plates, but was a concentration that might be expected to occur in the groundwater of soils following the agronomic application of the pesticide. After application, the treated and untreated nematodes were allowed to migrate on the chemotaxis plates for 1 h, at which time the numbers in each zone were counted. In either case approximately 50% of the *C. elegans* migrated out of zone A and into either zone B or C. In the assays the mean number of nematodes migrating towards the NaCl attractant in zone B was consistently and significantly (*P* < 0.05 by one way ANOVA and 2 sample t test) higher when using the untreated control nematodes as compared to when those treated with thiacloprid were applied to the chemotaxis plates. Once this had been determined, for both thiacloprid-treated and untreated nematodes replicate chemotaxis assay measurements were then used to calculate chemotaxis index mid-point and range values at the 95% confidence level (Fig. 1). The mid-point chemotaxis index of +0.48 for the untreated control group of nematodes indicated that in the absence of thiacloprid the majority of the nematodes that migrated out of zone A were attracted to and moved towards the NaCl spot in zone B. For the nematodes treated with 10 ng mL^−1^ thiacloprid the mid-point chemotaxis index of −0.0043 indicated that the pesticide treatment had effectively negated the ability of the nematodes to discern and respond to the presence of the NaCl attractant, such that their choice of direction of movement became considerably more random.

### Thiacloprid treatment of *C. elegans* prevented normal feeding-associated locomotion during NaCl chemosensing

When placed on agar plates, in the absence of an attractant, such as either a food source or NaCl, *C. elegans* demonstrate a pirouetting behaviour during their locomotion where they make frequent turns of ≥90° and thus, overall, appear to move in a directionless and random manner. However, once a positive chemo-attractant has been detected, the nematodes generally move along the chemical stimulus gradient, make fewer turns of this magnitude and thus, overall, appear to move in a determined direction towards the attractant.

**Fig. 3:**
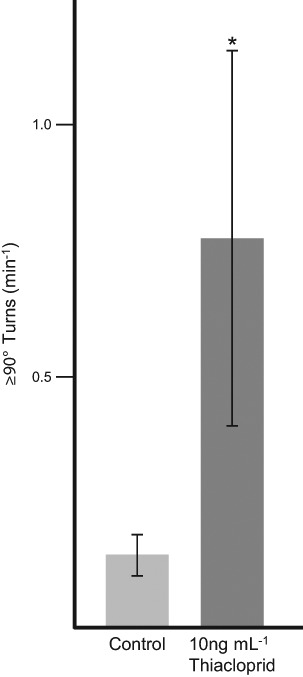
Thiacloprid altered the pirouetting behaviour of *C. elegans* during salt chemosensing. Six replicate samples of mixed growth stage populations of ≈150 *C. elegans* were treated +/−10 ng mL^−1^ thiacloprid for 1 h at 21 °C in S basal buffer before being applied to individual chemotaxis plates. During their subsequent 1 h migration on the plates the nematodes left visible tracks which were recorded photographically and the total number of turns of ≥90° which were made during the incubation period were recorded. The results are presented as the mean number of such turns made min^−1^ with the errors shown as the 95% confidence intervals around the calculated means in each case. *Indicates a significant difference in the rate of turning compared to the control (*P* < 0.05 by Dunn’s test)

Here, during the migration of the thiacloprid-treated and untreated nematodes across separate NaCl chemotaxis plates the movement tracks made by the *C. elegans* during the 1 h incubation period were recorded photographically. In each case the mean number of turns of ≥90° made by the nematodes was determined and is shown in Fig. 3. Nematodes exposed to 10 ng mL^−1^ thiacloprid for 1 h subsequently exhibited a significantly higher mean number of turns of this magnitude when placed on chemotaxis plates using a NaCl spot as the attractant, effectively performing 6 times more pirouettes than those of the untreated control groups. Thus, the indication is that the thiacloprid treatment effectively prevented the nematodes from perceiving the chemo-attractant and thus, from moving in a determined direction towards the source of the potential attraction.

### Sequence conservation of the ligand binding domains of the nAChRs of neonicotinoid-susceptible species

**Fig. 4:**
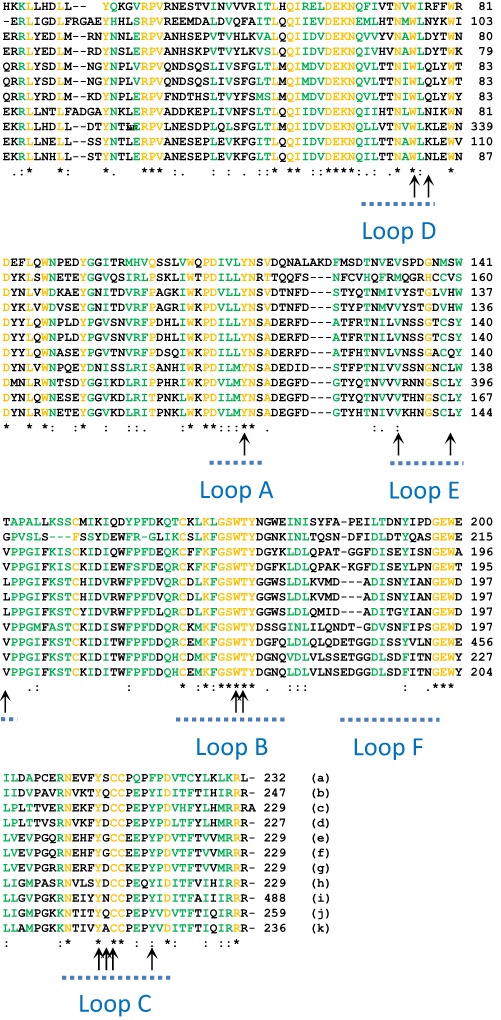
Clustal Omega amino acid sequence alignment of the ligand binding domains of neuronal nicotinic acetylcholine receptors (nAChRs) from thiacloprid-susceptible species. The Blastp alogorithm was used to identify amino acid sequences from the representative species indicated [(**a**) *P. dumerilii* (ACI88788), (**b**) *C. teleta* (ELU07054), (**d**) *H. contortus* (ABW07339), (**e**) *O. niloticus* (XP_003450802), (**f**) *H. burtoni* (XP_005930510), (**g**) *D. rerio* (AAI62599), (**h**) *H. rubusta* (ESN90492), (**i**) *M. domestica* (ABY40460), (**j**) *A. Melifera* (XP_006559134), (**k**) *D. melanogaster* (NP_001260302)] that showed a high degree of similarity to that of (**c**), the ligand-binding domain of the *C. elegans* nAChR-α16A subunit (NP_505207). The identified sequences were aligned using the multi-alignment programme Clustal Omega at EMBL-EBI. Invariant^(^*^)^ amino acids are highlighted in *orange* and substituted^(. or:)^ amino acids in *green*. *Arrows* indicate the amino acids thought to be involved in thiacloprid binding to the nAChRs of the honey bee (*A. melifera*) by either hydrogen bonding or hydrophobic interaction (Selvam et al. 2014)

**Fig. 5:**
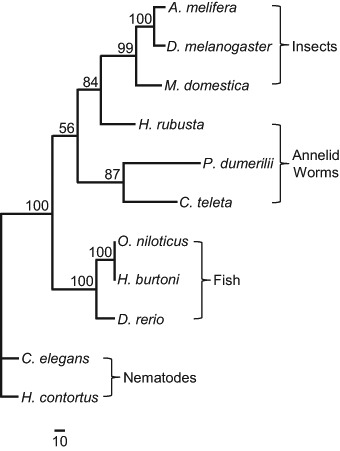
Neighbour joining phylogenetic tree analysis of the ligand binding domain amino acid sequences of neuronal nicotinic acetylcholine receptors (nACRs) from thiacloprid-susceptible species. The BlastP alogorithm was used to identify amino acid sequences from the representative species indicated [*A. melifera* (XP_006559134), *D. melanogaster* (NP_001260302), *M. domestica* (ABY40460), *H. rubusta* (ESN90492), *P. dumerilii* (ACI88788), *C. teleta* (ELU07054), *O. niloticus* (XP_003450802), *H. burtoni* (XP_005930510), *D. rerio* (AAI62599), *H. contortus* (ABW07339)] that showed a high degree of similarity to that of the ligand binding domain of the *C. elegans* nAChR-α16A subunit (NP_505207). Phylogenetic tree construction using the identified sequences was performed using Paup 4.0b10 (Swofford 2002). Bootstrap values after 1000 replicates are shown as percentages and the scale bar indicates the fraction of substitutions *per* amino acid site

Neonicotinoids effect their action through binding to the ligand binding domains of the neuronal nicotinic acetylcholine receptors (nAChRs) of susceptible species. Since *C. elegans* was obviously affected by thiacloprid, Clustal Omega amino acid sequence alignment and Paup 4 phylogenetic analyses were performed using the amino acid sequence of the ligand binding domain of its nAChR-α16A subunit and those of the most similar nAChR subunits identified for a number of other neonicotinoid-susceptible species by Blastp searching of the non-redundant sequences database at the NCBI (Figs. 4 and 5). Insects, fish, annelid worms and other nematodes have all previously been shown to be susceptible to neonicotinoids and comparison of the amino acid sequences of the ligand binding domains of the identified nAChR subunits indicated a high degree of phylogentic conservation between *C. elegans* and these species, especially in the amino acids considered to be involved in the binding of thiacloprid to insect nAChRs, as indicated by the arrows shown in Fig. 4.

### Development of *C. elegans* in the presence of thiacloprid increased expression of genes involved in apoptosis and cell secretion

**Fig. 6:**
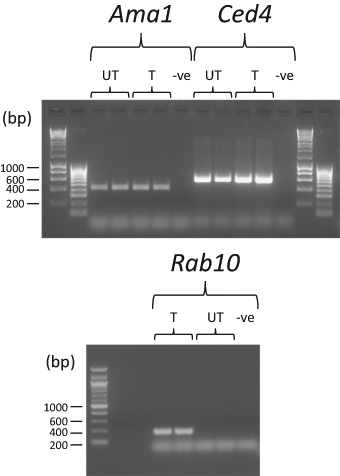
Accumulation of mRNAs encoding cell death and secretion related proteins in *C. elegans* following their exposure to the neonicotinoid pesticide, thiacloprid. Multiple thousands of *C. elegans* eggs were hatched on NGM plates in the absence of food and allowed to developmentally synchronise to L1 stage larvae for 24 h. Synchronised L1 larvae were then transferred *en masse* to *E. coli* OP50 lawns on NGM agar plates +/−10 ng mL^−1^ thiacloprid (UT-untreated; T-treated) and were allowed to develop at 21 °C to young adult stage, at which point total RNA was extracted and 8 µg aliquots were subjected to RT-PCR analysis with gene specific primers designed to amplify regions of the coding sequences for the proteins RNA polymerase I (Ama-1), Cell death protein 4 (Ced-4) and Ras-related protein 10 (Rab-10). Shown are the PCR products of duplicate experiments visualised by UV/EtBr staining after their electrophoresis in agarose gels alongside DNA size markers

Treatments that induce aberrant neural responses in *C. elegans* have previously been shown to be accompanied by neurodegeneration and increased neural apoptosis and cell secretion as a result of upregulated endocytic recycling (Wang and Audhya 2014). Thus, in order to determine if such processes resulted in *C. elegans* following their exposure to groundwater levels of thiacloprid, duplicate populations of developmentally synchronised nematodes were allowed to develop to the young adult stage in the presence of a bacterial food source +/−10 ng mL^−1^ thiacloprid and the expression of the genes *Cell death 4* (*Ced-4*) and *Ras-related protein 10* (*Rab-10*) was investigated by RT-PCR (Fig. 6). Expression of the constitutively expressed gene encoding RNA polymerase II (*Ama-1*) was also determined as a cDNA synthesis control to show both technical and RT consistency throughout.

While the expression of *Ama-1* appeared consistent throughout the experiment, the expression of *Rab-10* appeared to be greatly increased in the nematodes that had been cultured to young adult stage in the presence of 10 ng mL^−1^ thiacloprid. In the same *C. elegans* cultures, *Ced-4* expression was apparent at reasonable levels in both the untreated and thiacloprid-treated nematodes, but showed a marginal increase in those cultured in the presence of the neonicotinoid.

## Discussion

We observed that 10 ng mL^−1^ thiacloprid caused sufficient hyper-stimulation of the chemosensory neurons of the nematodes to the extent that they effectively chemo-sensed equally regardless of whichever direction they turned. In contrast with the untreated controls, the increased pirouetting of the nematodes, their random direction of movement and their inability to migrate positively towards the NaCl chemo-attractant after thiacloprid exposure was highly indicative of hyper-stimulation. *C. elegans* sense their environment primarily by using their amphid chemosensory organs, which contain eleven pairs of chemosensory neurons (Bargmann 2006). Proper functioning of these neurological pathways is important if the nematodes are to appropriately respond to chemosensory cues which, depending on the signal, can elicit chemotaxis, rapid avoidance, changes in overall motility and entry into and exit from the alternative, starvation-associated, stationary, dauer developmental stage.

Increased endocytic recycling in *C. elegans* is a process associated with numerous disease states, including neurodegeneration (Wang and Audhya 2014). Previous studies have shown a marked increase in the number of recycling endosomes during the onset of apoptotic neuro-degeneration in *C. elegans*, evidenced by an increase in Rab-11 expression (Troulinaki and Tavernarakis 2012), a GTPase that performs a similar role in endocytosis to that of Rab-10. Here, although the level of expression of the apoptosis-associated gene, *Ced-4*, showed only a marginal increase in the nematodes cultured to adult stage in the presence of thiacloprid, the very marked increase in the level of expression of *Rab-10* was indicative of enhanced endocytic processes that may have been associated with neurological dysfunction. The low level of thiacloprid used here was sufficiently toxic to enhance endocytic recycling in an attempt to replace dysfunctional nAChRs.

The argument that neonicotinoids, such as thiacloprid, are selectively toxic towards insects because of differences in the structure of their nAChRs may hold true when comparisons are made with mammals (Tomizawa and Casida 2005; Liu et al. 2010). However, it is fairly clear from this study that such an argument is less applicable when comparing insect nAChRs with those from other non-mammalian, neonicotinoid susceptible species. Phylogenetically, insects, annelid worms, fish and nematodes appear to separate into distinct clades when comparing the amino acid sequences of the ligand binding domains of their nAChRs, with the nematodes being furthest away from the insects in this comparison. However, the phylogenetic distances between the groups in the comparison presented here are not large and simple multiple sequence alignment showed how similar this domain is between these species and that, in the comparison made, there was crucially a high degree of conservation of the amino acids thought to be involved in binding of thiacloprid to the receptors of insects, such as the honey bee (Selvam et al. 2014). Neonicotinoids have previously been shown to be toxic to all the other species used to compare with *C. elegans* in this study (Gibbons et al. 2015).

Soil fauna such as nematodes play an important role as detrivores, recycling nutrients from the bacterial biomass (Bongers and Bongers 1998) in a manner that has been shown to be beneficial to plant growth (Ferris et al. 2012). Disrupting the chemosensory ability of such organisms may then have implications for soil ecology that impact negatively on agricultural practices that use organic matter as a source of plant nutrition. In our study, nematodes were provided with an abundance of bacterial food and realistically did not have to search for it. Thus, at the low level of thiacloprid used, development of the nematodes from L1 to the adult stage appeared to be visibly unaffected. However, it is less clear whether or not this would be the case in a field situation where the nematodes had to employ their chemosensory ability to find such food. Certainly, the feeding behaviour of other soil organisms, such as earthworms, has been shown to be negatively affected by the application of neonicotinoids in a field environment (Lal et al. 2001; Dittbrenner et al. 2010). *C. elegans* also use their chemosensory ability to avoid potentially harmful environments and to decide whether or not to enter the resting, dauer developmental stage in times of food deprivation. Should residual levels of neonicotinoids in the soil perturb such processes it would seem inevitable that some negative impact on nematode populations would occur as a consequence.

The neonicotinoid class of pesticides became available in the early 1990s with the commercial release of imidacloprid and because of the ease of their agronomic deployment, cost efficiency and their selectivity in controlling insect pests, they saw a rapid rise in their use worldwide. By 2010 neonicotinoids had attained a 26% share of the global market (Casida and Durkin 2013) with some 1.2 million hectares of crops being treated with neonicotinoids in the UK alone (Easton and Goulson 2013). Thiacloprid was released for agronomic use in 2000 (Elbert et al. 2000) and with its three orders of magnitude higher LD_50_ of 14.6 µg *per* bee, as compared to 18 ng *per* bee for imidacloprid (Iwasa et al. 2004), it escaped the temporary ban imposed on the use of other more potent neonicotinoids by the EFSA in 2013, which was enforced in order to allow time for research concerning their possible involvement in bee colony collapse disorder. While there is substantial evidence, well reviewed by Van der Sluijs et al. (2013), that neonicotinoids are harmful to bee populations, there has been far less consideration given to the wider off-target effects of these pesticides, in particular with regard to the potentially harmful effects of the less potent neonicotinoids on non-insect, invertebrate species. Here, the results presented indicated that relatively low levels of one of the less toxic neonicotinoids, thiacloprid, at sub-lethal concentrations that may be expected to occur in groundwater run-off, had potentially detrimental effects on the chemosensory ability of the soil nematode, *C. elegans*. It is thus, an ecological imperative that groundwater concentrations of even this less toxic neonicotinoid are not permitted to increase beyond those already apparent and it would be appropriate to investigate further the long term ecological impact of its use in the field.

## Conclusions

The environmental and ecological impact of the widely used neonicotinoid insecticides is currently poorly understood. This paper adds to growing evidence that their use may have persistent, unforeseen effects on non-target organisms. Here, specifically, thiacloprid was seen to impede the ability of *C. elegans* to engage in positive chemotaxis which coincided with a significant change in their normal locomotion characteristics.
